# Multilevel-3D Bit Patterned Magnetic Media with 8 Signal Levels Per Nanocolumn

**DOI:** 10.1371/journal.pone.0040134

**Published:** 2012-07-10

**Authors:** Nissim Amos, John Butler, Beomseop Lee, Meir H. Shachar, Bing Hu, Yuan Tian, Jeongmin Hong, Davil Garcia, Rabee M. Ikkawi, Robert C. Haddon, Dmitri Litvinov, Sakhrat Khizroev

**Affiliations:** 1 Department of Electrical Engineering, University of California Riverside, Riverside, California, United States of America; 2 Department of Computer Science & Engineering, University of California Riverside, Riverside, California, United States of America; 3 Department of Electrical Engineering, Florida International University, Miami, Florida, United States of America; 4 Department of Materials Science & Engineering, University of California Riverside, Riverside, California, United States of America; 5 Center for Nanoscale Science and Engineering, University of California Riverside, Riverside, California, United States of America; 6 Center for Nanomagnetic Systems, University of Houston, Houston, Texas, United States of America; University of Akron, United States of America

## Abstract

This letter presents an experimental study that shows that a 3^rd^ physical dimension may be used to further increase information packing density in magnetic storage devices. We demonstrate the feasibility of at least quadrupling the magnetic states of magnetic-based data storage devices by recording and reading information from nanopillars with three magnetically-decoupled layers. Magneto-optical Kerr effect microscopy and magnetic force microscopy analysis show that both continuous (thin film) and patterned triple-stack magnetic media can generate eight magnetically-stable states. This is in comparison to only two states in conventional magnetic recording. Our work further reveals that ferromagnetic interaction between magnetic layers can be reduced by combining Co/Pt and Co/Pd multilayers media. Finally, we are showing for the first time an MFM image of multilevel-3D bit patterned media with 8 discrete signal levels.

## Introduction

Presently, all magnetic-based data storage technologies are two-dimensional (2D) restricted. Conventional data storage devices store information in the form of 0′s and 1′s and are coded by the magnetization of ∼35 nanoscale magnetic grains. The magnetization of these grains is oriented in either of two possible states and depends on the media predefined magnetic anisotropy axis. In this way, the recording and reading are done in a binary fashion from a single continuous magnetic layer. Increasing the areal density of data storage is achieved by writing more bits per track and more tracks per radial distance. Therefore, all the main components (reader/writer elements and magnetic media grain size) of a hard disk drive (HDD) must scale down in order to “squeeze” more information per unit area. Tremendous progress has been made for about five decades by scaling these components down. Nonetheless, in the past decade, the longitudinal magnetic recording (LMR) technology reached a fundamental (superparamagnetic) limit for areal densities between 100–200 Gbit/in^2^ [1], [2].

The superparamagnetic limit occurs when thermal fluctuations in the media spontaneously switch the polarization of recorded bits within a relatively short time. This causes data loss and is an undesirable effect. The superparamagnetic limit is dependent upon the media grain size and magnetic anisotropy [3]. Hence, to defer this fundamental limit either higher anisotropy media or larger effective grain volume must be implemented. While the first requires relatively large switching magnetic field, the latter necessitates either thicker media or physical bit separation via patterning of the magnetic media.

To further increase the data storage capacity of HDDs, perpendicular magnetic recording (PMR) has been incorporated as a short-term solution. As the name suggests, bits are oriented in a perpendicular (out-of-plane) direction as opposed to the longitudinal (in-plane) direction. This enables magnetic recording on larger anisotropy magnetic media with greater grain volume [4]. As with LMR, limitations of PMR include media stability and read/write field. Since PMR is predicted to reach the superparamagnetic limit at ∼1 Tbit/in^2^, alternate technologies are vital to advance the magnetic-based storage technology and save it from becoming a mere commodity.

The two main proposed technologies under serious consideration are heat (or energy) assisted magnetic recording (HAMR) and bit patterned media (BPM). In the case of HAMR, a hybrid recording head is used to provide both heat and magnetic energy to switch the magnetization of ultrahigh anisotropy magnetic media [5], [6]. Main obstacles for this technology include allocation of a nanoscale heat spot with sufficient energy and fabrication of suitable magnetic media with sub-5 nm grain size [7], [8]. The BPM approach can defer the superparamagnetic limit by physically patterning the magnetic media [9]. Since the media is composed of exchange-coupled magnetic grains, the effective volume of a patterned bit is attributed to the volume of the bit as opposed to the volume of a single grain within a bit. Consequently, the bit becomes resilient to thermal fluctuations and the readback signal-to-noise ratio (SNR) increases as well. For fabrication, either direct patterning of the magnetic media or deposition of the magnetic composition on prepatterned media can be employed. Issues that must be addressed to achieve this technology include the mass production of patterned disks with bit separation below 25 nm and read/write position synchronization [10]. Regardless of the implementation level of either or both technologies, they are still 2-D limited. Eventually, multilevel (ML)-3D magnetic recording may be obligatory to further advance the progression of higher density magnetic storage [11], [12].

**Figure 1:**
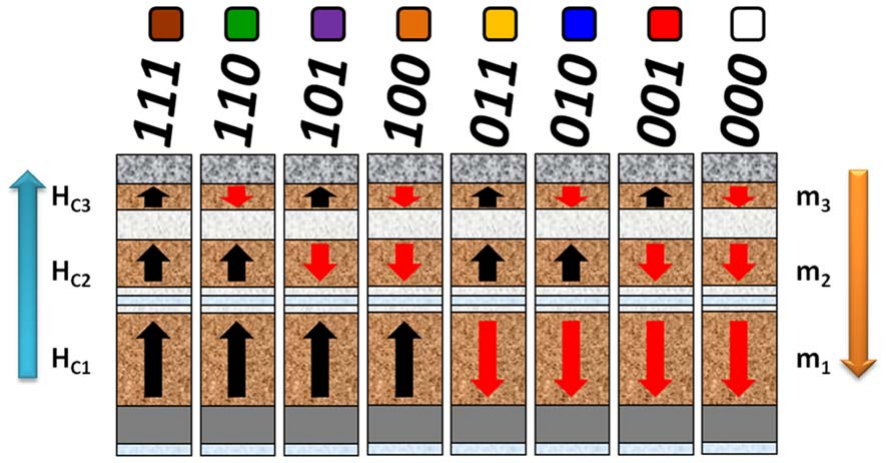
Triple-stack ML-3D BPM. Schematic of the structure and desired magnetic properties (not to scale). The black arrows (binary “1″) and red arrows (binary “0″) denote the two possible magnetic states in each stack. Above the columns lies the binary representation and color coding for the eight possible magnetic states.

In ML-3D magnetic recording, at least two magnetic layers (*N* ≥2) are used to both read and write information. The ML readback signal is proportional to the vector sum of the magnetic fields generated by the *N* magnetic layers. Amplitude detection of the playback signal is thus required to distinguish the various signal levels. Such detection mechanism has been implemented previously in ML optical storage [13], [14]. Assuming an ideal case where each layer generates two readable and distinguishable signal levels, the effective areal density increases by a factor of *N*. To selectively write on *N* layers, the ML-3D magnetic media must exhibit a variation in magnetic properties across its thickness. One option is to create a coercivity (*H_cN_*) gradient, where individual magnetic layers are accessed by changing the magnitude and direction of the write field. Finally, integrating the ML-3D approach with BPM may also relax the patterning constraints while still increasing the capacity of data storage devices. This is because the effective information density becomes a function of the number of signal levels that can be accessed as well as the distance between individual bits.

**Figure 2:**
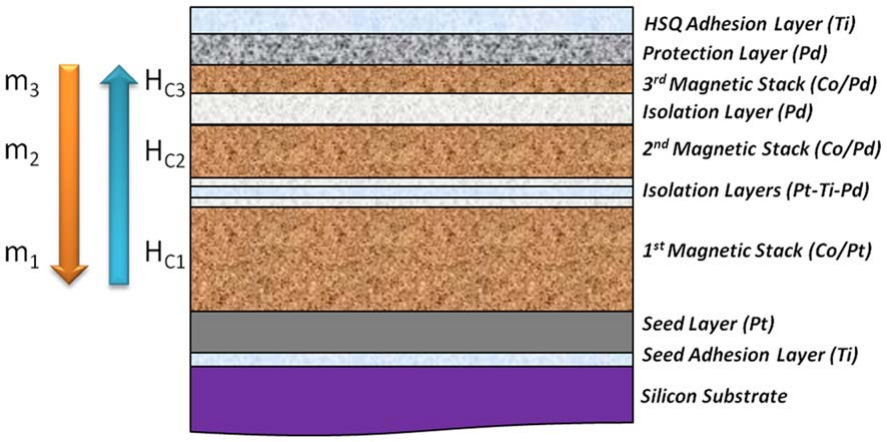
ML-3D magnetic media composition.

To date, both industry and academic institution have showed growing interest in ML-3D magnetic recording [15], [16], [17], [18]. Feasibility work has been presented on dual-layer Co/Pd and dual-layer Co/Pt multilayers [19], [20]. The main concern under consideration was the ferromagnetic coupling between multilayers stacks of Co/Pd-Co/Pd and Co/Pt-Co/Pt dual-stack media [21]. It was shown that opposing poles were not the preferred demagnetization state for these types of media. Alternate magnetic dual-layer media using oblique evaporated Co-CoO medium has also been proposed as a method of recording on more than one layer [22]. Finally, implementation of microwave assisted magnetic recording was also considered for dual-layer ML-3D recording [23], [24].

Arguably, the most important challenges with addressing more than one magnetic layer can be summarized as the following three problems. First, the recording field is weaker at larger distances from the recording head. Therefore, a limitation exists on the total number of independent magnetic layers that can be stacked across the media. In order for a bit of information to be recorded, the magnetic field should overcome the coercivity of the media in the region of the bit. Second, it is a challenge to selectively write on each layer due to the relatively strong ferromagnetic interaction between adjacent layers. Third, it is not trivial to distinguish the magnetic state of individual magnetic layers during the playback process because of the superposition of all the fields emanating from different layers. To address these challenges and demonstrate the feasibility of a ML-3D recording system, we have studied hybrid ML-3D media that is composed of both Co/Pd and Co/Pt multilayers. In this letter, we reveal the results of triple-stack (Substrate)-Co/Pt-Co/Pd-Co/Pd ML-3D BPM. The analysis of this media highlights our findings and signifies the combination of Co/Pd and Co/Pt multilayers in the first generation ML-3D BPM.

**Figure 3:**
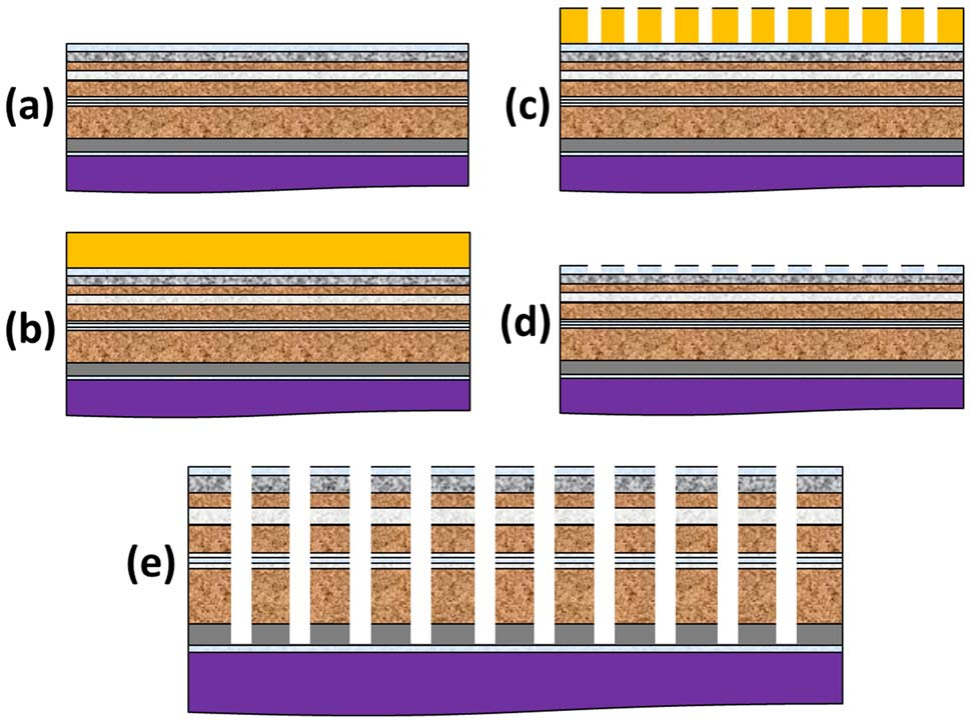
ML-3D BPM media fabrication process. (a) Sputter-deposition of the triple-stack ML-3D magnetic media composition (b) Spin-coat and bake HSQ (c) E-beam lithography (expose and develop patterns) (d) ICP etching of the naturally oxidized Ti layer (e) Ar-ion milling of the remaining composition to form the triple-stack ML-3D BPM.

We exploit exchange-coupled Co/Pd and Co/Pt multilayers, which due to their relatively high perpendicular magnetic anisotropy are being considered for PMR and BPM [25], [26]. The structure of multilayers media is composed of a stack of Co/Pt or Co/Pd pairs, where the thickness of each pair is commonly below ∼2 nm. Because of the relatively thin Pd or Pt thickness, the pairs within a single stack of multilayers are exchange-coupled. Consequently, each multilayers stack is considered to be a single magnetic layer. To isolate adjacent stacks for ML-3D magnetic recording, a relatively thick non-magnetic composition is inserted between the stacks.

To effectively fabricate ML-3D media, we amalgamate Co/Pd and Co/Pt multilayers to provide a substantially wider range of key properties, such as coercivity (*H_c_*) and saturation magnetization (*M_s_*). These properties can be controlled via the deposition parameters, selected compounds, thickness of each element within a pair, and the number of pairs within a stack [27]. For this study, a combinatorial synthesis process has been implemented to achieve material compositions necessary to control gradients of coercivity and magnetic moment (*m_N_*) across the thickness of ML-3D media. Varying the magnetic moment across the recording media makes it possible to distinguish different stacks from each other during the readback process. The *M_s_* and thickness of each stack define its total magnetic moment. In summary, we came up with the following rule of thumb to define relationships between the magnetic properties of different magnetic layers: *m_1_≥2m_2_≥4m_3_≥2^N−1^m_N_* and *H_cN_>H_c3_>H_c2_>H_c1_*, where *m_1_* and *H_c1_* are the total magnetic moment and coercivity of the bottommost layer. Figure 1 shows a schematic representation of the media structure and preferred magnetic properties. The attainable magnetic states and their binary and color notations are illustrated as well.

**Figure 4:**
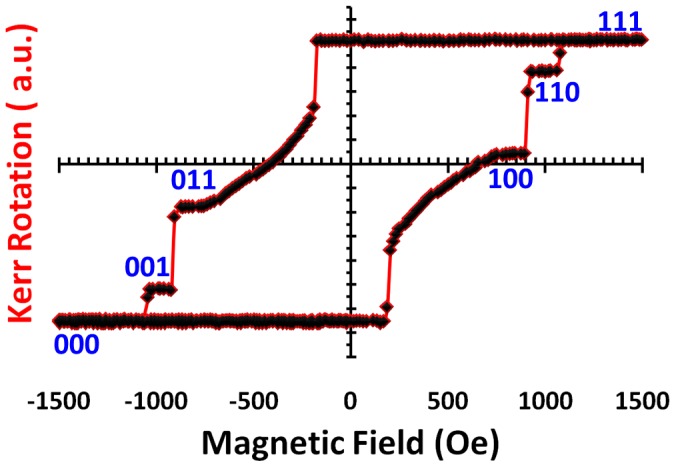
MOKE hysteresis loop. A graph revealing three magnetically-decoupled recording layers for the fabricated ML-3D media.

**Figure 5:**
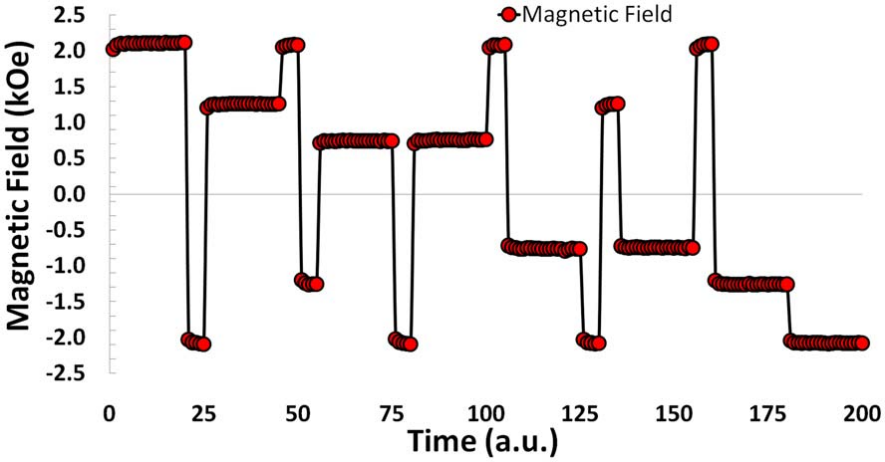
Modulated recording field. The sequence of magnetic fields applied to the triple-stack ML-3D media. The transitional external fields required to achieve the desired magnetic state were given 5 time units while 20 time units were designated for the final magnetic field.

**Figure 6:**
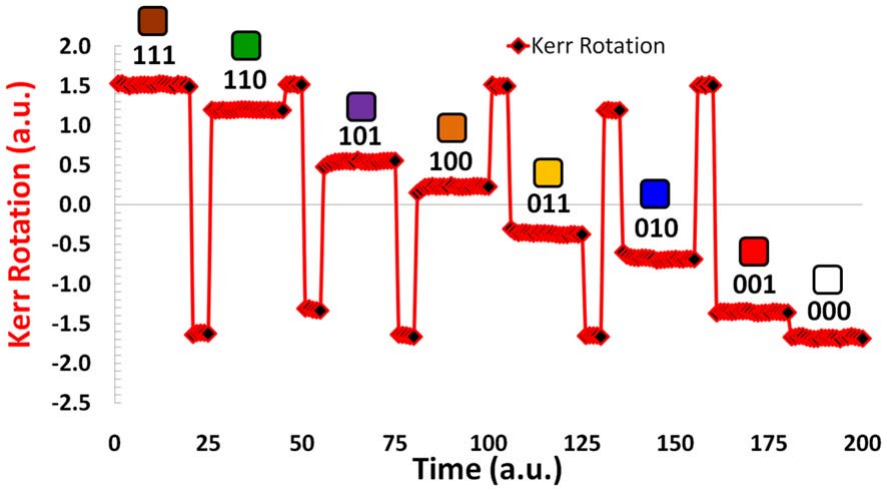
Kerr rotation measurement. The simultaneous Kerr rotation produced by the ML-3D media as a result of the externally applied magnetic fields. The binary representation for each level along with its color coding are listed above each of the eight magnetic states.

## Materials and Methods

### Homogeneous ML-3D Media Fabrication

The surface properties of a Si (100) substrate were modified in order to optimize the magnetic properties of the ML-3D media. This was done by Ar-ion milling the native SiO_2_ on the surface of the substrate prior to deposition. A selected composition of (Substrate)-Ti(1.5 nm)/Pt(5 nm)/[Co(0.4 nm)/Pt(0.55 nm)]×14/Pt(1.5 nm)/Ti(1.5 nm)/Pd(1.5 nm)/[Co(0.25 nm)/Pd(0.55 nm)]×7/Pd(4.5 nm)/[Co(0.25 nm)/Pd(0.55 nm)]×3/Pd(3 nm)/Ti(4 nm) was then sputter-deposited on the substrate, Figure 2. This composition was nominated to both demonstrate the feasibility of effectively storing magnetic information in a 3^rd^ dimension and enable MFM imaging of all the readback levels with acceptable SNR. For the presented ML-3D media, the effective magnetic moment in each strongly coupled multilayers stack was closely proportional to the total Co thickness in each layer. This was determined from a vibrating-sample-magnetometer (VSM) measurement. To ensure adequate decoupling between adjacent magnetic layers, we maintained an isolation thickness of ∼5 nm. The Ti interlayer (1.5 nm) between the Co/Pt and first Co/Pd multilayers stacks promoted exchange-coupled grains within the Co/Pd multilayers stacks. Following the deposition process, the media was placed in ambient conditions to induce the natural oxidation of the Ti capping layer. The oxidized Ti layer served as an HSQ adhesion layer as well as a hard-mask for the pattern transfer process, e.g. from HSQ nanoscale patterns to ML-3D magnetic nanopillars.

### Patterned ML-3D Media Fabrication

Electron-beam lithography (EBL) was used to fabricate arrays of periodic hydrogen silsesquioxane (HSQ) nanoscale patterns on the surface of the above described triple-stack ML-3D media. First, diluted HSQ and Methyl isobutyl ketone (MIBK) 1∶2.5 was spin-coated on the ML-3D magnetic media, followed by heat treatment at ∼140°C, Figure 3(b). Second, the patterns were exposed by a JEOL direct-write EBL system (JBX-5500FS) using 50 keV beam intensity, 98 pA beam current, and an average dose of 3000 µC/cm^2^. The sample was then developed in a MICROPOSIT™ MF™ CD-26 solution to uncover the electron-beam exposed HSQ patterns, Figure 3(c). The morphology and height of the developed patterns were confirmed with scanning electron microscopy (SEM) and AFM, respectively. A total of ∼30 nm was measured for the final thickness of the developed HSQ patterns. Implementation of the above process produced HSQ features below 10 nm in diameters with separation distance of ∼26 nm.

**Figure 7:**
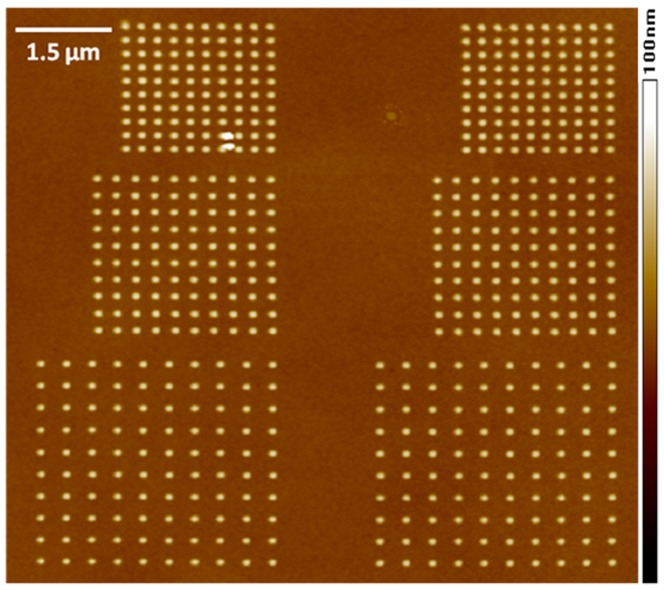
AFM image of the triple-stack ML-3D BPM.

The procedure of transferring the HSQ patterns into the media necessitated a couple of steps. First, we exposed the sample to inductively-coupled plasma (ICP) reactive etching using a gas mixture of SF_6_/Ar (50∶50) at ∼450 W for 30 seconds. The bare oxidized Ti layer was removed from around the HSQ patterns during this process. Second, the ICP power dropped to ∼250 W and the SF_6_ source closed, leaving only Ar gas in the chamber. This resulted in Ar-ion milling (or sputtering) of the HSQ patterns and ML-3D composition, which continued for an additional six minutes, Figure 3(e). The Ar-ion milling parameters were set to virtually not affect the oxidized Ti layer. Finally, confirmation of the transferred patterns was done through SEM and AFM imaging.

### Magnetic Writing and Reading via Magnetic Force Microscopy

High resolution MFM probes with relatively high *M_s_*, magnetic moment, and coercivity were custom designed and fabricated by Paramount Sensors LLC for this project. These probes were used to both write and read from the ML-3D BPM. To facilitate adequate writing and reading, these probes needed to exhibit a range of magnetic fields. Strong fields were needed to magnetize the media for writing while weaker fields were required to ensure no significant magnetic switching occurred during the MFM reading process. Fabricating the probes with a range of different protective coating thicknesses accomplished this. The probes with the thinner protective coating produced the highest magnetic field in the vicinity of the tip and were used to switch the magnetization of the Co/Pt stack in selected nanopillars. On the other hand, the probes with the relatively thicker protective coating emanated weaker magnetic fields and were utilized to read the recorded information. MFM imaging of the nanopillars was implemented using the *Lift Mode™* technique with a tip-sample separation of ∼20 nm.

## Results and Discussion

### Homogeneous ML-3D Media

The out-of-plane hysteresis loop for the entire ML-3D media composition is shown in Figure 3. Measurements of the curve were taken using a custom-built polar magneto-optical Kerr effect (P-MOKE) system. The discontinuous shape of the resulting hysteresis loop proves that the three stacks (each being a set of strongly coupled multilayers) are exchange-decoupled from each other. Also evident from this loop, are the magnetic moment and coercivity gradients. This means that the three magnetic layers (stacks) can be separated from each other during write and read operations. Furthermore, a carful inspection of the hysteresis curve reveals that the bottommost (Co/Pt) and topmost (Co/Pd) layers produced Kerr signals which are relatively lower and higher when compared to the total Co thickness in each layer, respectively. This is the result of light transmission/reflection from the triple-stack media. The MFM interaction with the triple-stack media can thus be approximated from the hysteresis measurement. In MFM analysis, the proximity of the magnetic tip with respect to each magnetic layer will produce a similar outcome.

**Figure 8:**
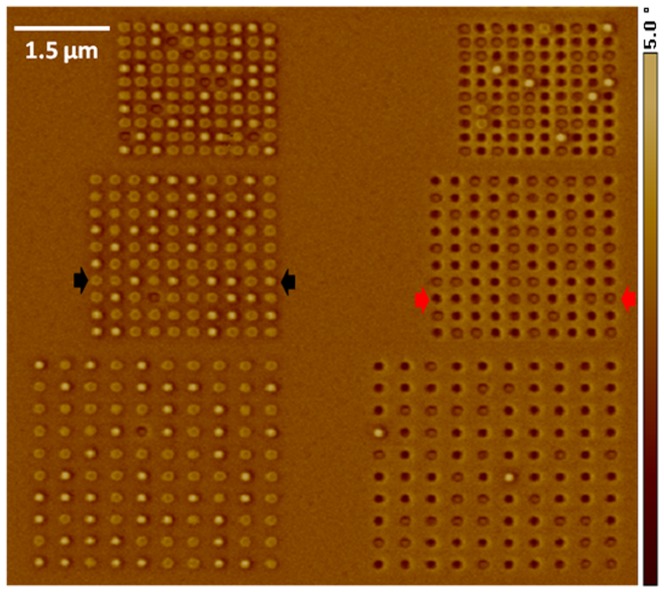
MFM image of the triple-stack ML-3D BPM. The MFM image corresponds directly to the nanopillars shown in the Figure 7.

**Figure 9:**
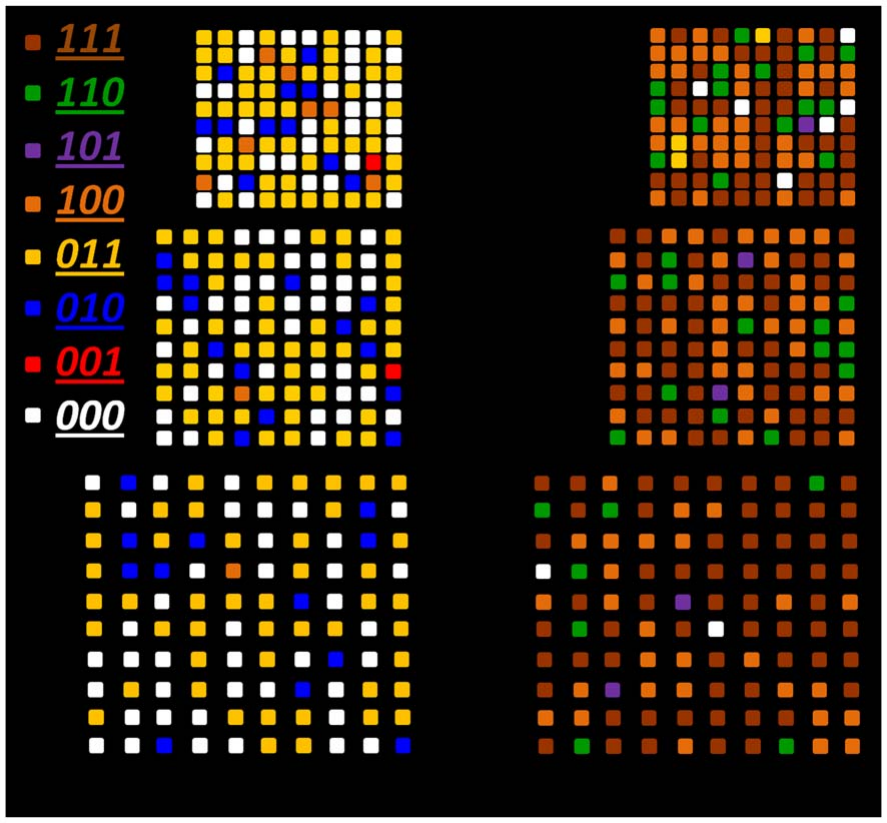
Diagram of MFM data. A schematic highlighting the various magnetic states of the nanopillars shown in Figure 7.

**Figure 10:**
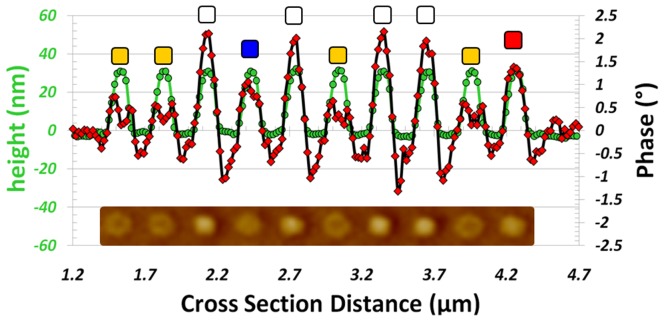
Quantification of ML MFM signals. AFM/MFM section line analysis of the row of nanopillars located between the black arrows in Figure 8. The colored squares represent the different magnetic states as shown in Figure 1. The inset is a cropped section of the MFM image, taken between the two black arrows.

**Figure 11:**
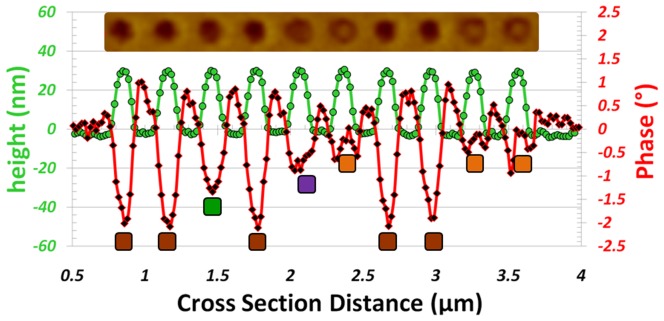
Quantification of ML MFM signals. AFM/MFM section line analysis of the row of nanopillars located between the red arrows in Figure 8. The color boxes represent the different magnetic states as shown in Figure 1. The inset is a cropped section of the MFM image, taken between the two red arrows.

To write 8-level information in the triple-stack ML-3D media, we take advantage of the knowledge stored in the hysteresis loop. The full hysteresis loop is defined as the reaction of the magnetization to continuous variation of externally applied magnetic field, from +∞ to -∞ and back to +∞. Using the full loop ensures a predictable way to record six of the eight signal levels (i.e. 111, 011, 001, 000, 100, and 110) in no more than two steps, Figure 4. To record these six levels, the ML-3D media is magnetized in a certain order according to the full loop. For example, to achieve the signal level “011”, a large positive recording field is applied as the first step to saturate the media in “111” direction, and as the second step, the field is reversed to the value sufficient to reach the desired magnetic state. The same way, any other of the six states can be reached. To attain the two remaining states (101 and 010), it is necessary to use minor hysteresis loops and thus three steps to perform the recording. To initiate a minor hysteresis loop, a field reversal has to be initiated before the full loop is closed. Figure 5 and Figure 6 show how all eight levels can be recorded (via externally applied magnetic fields) and simultaneously read (via the P-MOKE) from the triple-stack ML-3D media, respectively. The short time slots reflect transitional external fields which are applied to achieve the desired magnetic state. The longer time periods indicate the final externally applied magnetic field to achieve each of the eight magnetic states.

### Patterned ML-3D Media

The ML-3D BPM was initially demagnetized to investigate the preferred magnetic energy state of the nanopillars. To perform this analysis, the sample was exposed to an alternating and decaying magnetic field, starting at ∼7,500 Oe and ending at ∼1 Oe. The magnetic field alternated along the easy axis of the magnetic media (out-of-plane direction). We then analyzed the demagnetized media by performing MFM imaging on arrays of nanopillars. The MFM data showed that more than 97% of the nanopillars were in a partially antiferromagnetic state (e.g. 100 or 011 states). These results revealed that for this particular ML-3D BPM, the stored information across each nanopillar would be stable for at least two states with undesirable (antiferromagnetic) orientations.

Ideally, a nanoscale electromagnetic transducer would have been used to selectively write on each layer of each nanopillar by locally varying the magnitude and direction of the write field. In the present case, we configured the ML-3D BPM to exhibit 8-level magnetic information by further exposing it to a series of externally applied magnetic fields. To start with, a magnetic field of ∼17,000 Oe was applied along the magnetic hard axis (in-plane direction) of the media. Applying this field facilitated partial recording of the antiferromagnetic states between the two Co/Pd stacks (e.g. X01 and X10, where “X” represents the magnetic state of the bottommost Co/Pt layer which may either be a “0” or a “1”). Approximately 13% of the analyzed nanopillars changed their magnetic states from “X00” or “X11” to “X01” or “X10”. At this stage, the four possible magnetic states (e.g. X11, X00, X01, and X10) for the two Co/Pd stacks were locked in place. Randomizing the magnetic state of the third Co/Pt stack would finalize the recording of all eight levels. This could be done without modifying the previously recorded states in the Co/Pd stacks since the switching field for the Co/Pt stack was less than the coercivity fields of the Co/Pd stacks. First, we applied a magnetic field of ∼3,000 Oe along the magnetic easy axis of the media. This field caused the magnetic state of the Co/Pt stack in each nanopillar to be in the “0” state. Next, an MFM probe with the thinnest protective coating was magnetized in such a way that when used to AFM-scan the nanopillars, the magnetization of the Co/Pt stack in each nanopillar would switch its state from “0” to “1”. The set of patterns on the right-side of Figure 7 was AFM-scanned with this MFM probe. Finally, the eight magnetic states of the ML-3D BPM could be simultaneously revealed by taking an MFM image of both sets of patterns.

Magnetic force microscopy imaging of the nanopillars shown in Figure 7 was taken using an MFM probe with relatively thicker protective coating. Prior to imaging, the probe was magnetized along the tip central axis and into the tip (in the “1” direction with respect to the media). This probe caused the Co/Pt stack of only several nanopillars to switch its magnetic state from “0” to “1” during the initial scan of the patterns. Therefore, the protective coating thickness for this particular probe proved sufficient to enable the MFM reading of the final magnetic states.

The MFM image shown in Figure 8 was captured after a few scans of the same sets of patterns. The darker and lighter colors of the image-scale indicate the level of long-range magnetostatic attraction and repulsion between the tip and media, respectively. Therefore, the nanopillars that generated the lowest MFM signal (dark brown) were in the “111” state, while the ones that produced the highest signal (bright yellow) were in the “000” state. An assessment of the left-side set of patterns indicated that the globally applied magnetic field of ∼3,000 Oe switched the magnetization of the Co/Pt stack to the “0” state. It also showed the location of the nanopillars that had their magnetic state modified in the first MFM scan. The results for the patterns on the right-side revealed that the MFM probe with the thinnest protective layer generated strong enough magnetic field to change the magnetic state of the bottommost layer in more than 97% of the nanopillars.

A detailed examination of Figure 8 exposed all the possible magnetic states for the triple-stack ML-3D BPM. To highlight the various magnetic signal levels, we examined the MFM signal produced by each nanopillar and created a diagram representation for the MFM image, Figure 9. These states were quantified by performing AFM/MFM section line analysis across the central points of the nanopillars. Figure 10 and Figure 11 are examples of section line investigations, which were performed for the rows of nanopillars located between the black and red arrows in Figure 8, respectively. As is evident from the diagram and section line investigations, the triple-stack ML-3D BPM produced eight discrete magnetic states.

### Conclusion

ML-3D BPM has been designed and fabricated to effectively store eight levels of magnetic information. We show the principles of recording and reading information from a ML-3D recording system via externally applied magnetic fields, the magneto-optical Kerr effect, and magnetic force microscopy. The MFM measurements clearly show the discretization of the magnetic signals emanating from the ML-3D BPM. These signals are further proven to be repeatable and distinguishable throughout the analyzed patterns. Furthermore, we show that ferromagnetic interaction between adjacent magnetic layers can be significantly reduced by amalgamating Co/Pt with Co/Pd multilayers media.
